# Supplementary material for: Multifocal cutaneous neoplastic vascular proliferations in a rainbow boa (Epicrates cenchria) collection with boid inclusion body disease
Source: PLoS One. 2024 Nov 6;19(11):e0311015. doi: 10.1371/journal.pone.0311015 (PMC11540211; doi:10.1371/journal.pone.0311015)
Supplement: S1 Table — Examined cases, number and distribution of cutaneus lesions and tests performed. (DOCX) [file pone.0311015.s001.docx]

**Supplemental Table 1**. Examined cases, number and distribution of cutaneus lesions and tests performed.

| **Case No** | **Age (y)** | **Origin** | **Sex** | **Skin lesions^1^** | | | **Other pathological changes** | **In situ examinations^2^** | | **RT-PCR^5^** |
| --- | --- | --- | --- | --- | --- | --- | --- | --- | --- | --- |
|  |  |  |  | **V** | **L** | **D** |  | **IB^3^** | **IH^4^** |  |
| 1 | 13* | SP | M | 9 | 4 | - | NE | +  (Skin lesion) | +  (Skin lesion) | Pool^6^: pos |
| 2 | 13* | RJ | M | 8 | 2 | 1 | NE | +  (Skin lesion) | NE | Pool^6^: pos |
| 3^a^ | 15 | I | M | 17 | 5 | 2 | NE | -  (Skin lesion) | -  (Skin lesion) | Pool^6^: pos |
| 4^a^ | 15 | I | M | >20 | 3 | - | NE | +  (Skin lesion) | NE | Pool^6^: pos |
| 5^b^ | 13 | I | M | 7 | 3 | 1 | NE | +  (Skin lesion) | NE | Pool^6^: pos |
| 6^b^ | 13 | I | M | 6 | 1 | - | NE | -  (Skin lesion) | NE | Pool^6^: pos |
| 7 | 16* | BH | F | >20 | 6 | - | Liver: peliosis hepatis; severe hemosiderosis; moderate pyogranulomatous hepatitis | -  (Organs^7^) | -  (Organs^7^) | Neg^7^ |
| 8 | 15* | SP | M | >20 | 19 | 7 | Liver: peliosis hepatis; severe hemosiderosis | +  (Organs^8^) | +  (Organs^8^) | Neg^8^ |

Legend: BH – state of Bahia; F – female; M – male; I – internal (bred in-house); NE – not examined; pos – positive; ); RJ – state of Rio de Janeiro; SP – state of São Paulo; y – years (*implies that the animals were at least this age; they were brought into the collection as juveniles or young adults).

^a^These animals were siblings, from the same clutch; the parental animals (M03 and F01) were among the first rainbow boas brought in from externally to set up the colony. They have died in the meantime (no further information available).

^b^These animals are offspring from the same paternal animal (M02), with different maternal animals (F03 and F08, respectively). Also, these were among the first rainbow boas brought in from externally to set up the colony. They have died in the meantime (no further information available).

+ - IBs were detected in an HE stained tissue section and there was eveidence of reptarenavirus antigen expression by immunohistochemistry, respectively; - - IBs were not observed in an HE stained tissue section and there was no evidence of reptarenavirus antigen expression by immunohistochemistry, respectively.

^1^Distribution (D: dorsal; L: lateal; V: ventral) and number of skin lesions.

^2^Histological examination to classify the skin lesions and to identify any histopathological changes, combined with an immunohistochemical staining for reptarenavirus and hartmanivirus antigen.

^3^Presence of intractoplasmic inclusion bodies in skin lesions or organs.

^4^Detection of reptarenavirus antigen expression in skin lesions or organs (NB: There was no evidence of hartmanivirus antigen expression in any tissue.)

^5^Multiplex RT-PCR, following a previously published protocol (Baggio et al., 2023). The multiplex RT-PCR reaction was repeated on the purified PCR products obtained from an initial multiplex RT-PCR run. After agarose gel electophoresis, when present, bands at the corresponding expected size of reptarenavirus amplicons (approximately 150 bp) were excised and the amplicons were gel purified. These RT-PCR products were then Sanger sequenced and analysed through a BLAST search (<https://blast.ncbi.nlm.nih.gov/Blast.cgi>) to determine whether they corresponded to reptarenavirus sequences.

^6^Pool: RNA was extracted from a pool of small samples from 5 cutaneous lesions (from case nos. 1, 2, 5-7). After cDNA synthesis, multiplex RT-PCR and sequencing of gel purified PCR products were performed, confirming reptarenavirus infection (“pos”). PCR to detect Bartonella infection was performed on DNA extracted from the same pool of samples, following standard diagnostic protocols.

^7^Case 3: Liver, kidney, brain and heart were examined histologically and by IH. RNA extraction, cDNA synthesis and multiplex RT-PCR were undertaken on a liver biopsy; this did not yield a PCR amplicon of the expected size (“neg”).

^8^Case 4: Liver, brain, esophagus, stomach, small intestine, large intestine, spleen, pancreas, kidney, adrenal glands, testicles and epididymis were examined histologically and by immunohistology for reptarenavirus and hartmanivirus antigen. RNA isolation, cDNA synthesis and multiplex RT-PCR were undertaken on liver, kidney, testicle and a sample from a cutaneous lesion. Sequencing was performed on gel purified PCR products originating from the testicle sample. This did not yield a sequence consistent with reptatenavirus infection (“neg”).
